# Is there a role of whole-body bone scan in patients with esophageal squamous cell carcinoma

**DOI:** 10.1186/1471-2407-12-328

**Published:** 2012-08-01

**Authors:** Shau-Hsuan Li, Yung-Cheng Huang, Wan-Ting Huang, Wei-Che Lin, Chien-Ting Liu, Wan-Yu Tien, Hung-I Lu

**Affiliations:** 1 Department of Hematology-Oncology, Kaohsiung Chang Gung Memorial Hospital and Chang Gung University College of Medicine, Kaohsiung, Taiwan; 2 Department of Nuclear Medicine, Kaohsiung Chang Gung Memorial Hospital and Chang Gung University College of Medicine, Kaohsiung, Taiwan; 3 Department of Pathology, Kaohsiung Chang Gung Memorial Hospital and Chang Gung University College of Medicine, Kaohsiung, Taiwan; 4 Department of Diagnostic Radiology, Kaohsiung Chang Gung Memorial Hospital and Chang Gung University College of Medicine, Kaohsiung, Taiwan; 5 Department of Thoracic & Cardiovascular Surgery, Kaohsiung Chang Gung Memorial Hospital, Chang Gung University College of Medicine, Kaohsiung, Taiwan, 123 Ta-Pei Road, Niaosung Hsiang, Kaohsiung Hsien, Taiwan, ROC

**Keywords:** Radionuclide imaging, Esophageal cancer, Squamous cell carcinoma, Metastasis, Esophagectomy

## Abstract

**Background:**

Correct detection of bone metastases in patients with esophageal squamous cell carcinoma is pivotal for prognosis and selection of an appropriate treatment regimen. Whole-body bone scan for staging is not routinely recommended in patients with esophageal squamous cell carcinoma. The aim of this study was to investigate the role of bone scan in detecting bone metastases in patients with esophageal squamous cell carcinoma.

**Methods:**

We retrospectively evaluated the radiographic and scintigraphic images of 360 esophageal squamous cell carcinoma patients between 1999 and 2008. Of these 360 patients, 288 patients received bone scan during pretreatment staging, and sensitivity, specificity, positive predictive value, and negative predictive value of bone scan were determined. Of these 360 patients, surgery was performed in 161 patients including 119 patients with preoperative bone scan and 42 patients without preoperative bone scan. Among these 161 patients receiving surgery, 133 patients had stages II + III disease, including 99 patients with preoperative bone scan and 34 patients without preoperative bone scan. Bone recurrence-free survival and overall survival were compared in all 161 patients and 133 stages II + III patients, respectively.

**Results:**

The diagnostic performance for bone metastasis was as follows: sensitivity, 80%; specificity, 90.1%; positive predictive value, 43.5%; and negative predictive value, 97.9%. In all 161 patients receiving surgery, absence of preoperative bone scan was significantly associated with inferior bone recurrence-free survival (P = 0.009, univariately). In multivariate comparison, absence of preoperative bone scan (P = 0.012, odds ratio: 5.053) represented the independent adverse prognosticator for bone recurrence-free survival. In 133 stages II + III patients receiving surgery, absence of preoperative bone scan was significantly associated with inferior bone recurrence-free survival (P = 0.003, univariately) and overall survival (P = 0.037, univariately). In multivariate comparison, absence of preoperative bone scan was independently associated with inferior bone recurrence-free survival (P = 0.009, odds ratio: 5.832) and overall survival (P = 0.029, odds ratio: 1.603).

**Conclusions:**

Absence of preoperative bone scan was significantly associated with inferior bone recurrence-free survival, suggesting that whole-body bone scan should be performed before esophagectomy in patients with esophageal squamous cell carcinoma, especially in patients with advanced stages.

## Background

Esophageal cancer occurs worldwide. Esophageal cancer is the sixth most common cause of cancer death among men in Asian countries. Adenocarcinoma and squamous cell carcinoma are the major two histologic types of esophageal cancer. Although adenocarcinomas are more prevalent in the United States, [1] ninety percent of all esophageal cancers among Asian men are esophageal squamous cell carcinoma [1]. Despite advances in the diagnosis and treatment of esophageal cancer in recent decades, the prognosis of patients with esophageal squamous cell carcinoma still remains poor [2]. The 5-year survival of patients diagnosed with esophageal squamous cell carcinoma is around 30% [3]. Despite preoperative staging with contrast enhanced computed tomography and endoscopic ultrasonography, [4,5] patients continue to develop distant recurrences after esophagectomy [5-8]. The pattern of distant recurrence after esophagectomy has previously been described [6,7]. Bhansali et al. [7] reported that 19 (21%) of 95 patients with esophageal squamous cell carcinoma developed distant recurrence after esophagectomy, including 9 in the lung, 4 in the bone, 3 in the liver, 2 in the skin, and 1 in the brain. Lamb et al. [6] described that 28 (13.9%) of 201 patients developed distant recurrence after esophagectomy, predominantly isolated hepatic (11 patients) and bone (11 patients) metastases.

The role of whole-body bone scan has been elucidated in several cancers including breast cancer, [9] non-small cell lung cancer, [10] and prostate cancer[11]. However, the role of bone scan in patients with esophageal cancer remains controversial. Allum et al. [4] did not recommend bone scan as initial staging examination in current protocols. Quint et al. [12] suggested that routine preoperative bone scan identifies metastases only in the presence of widespread metastatic or unresectable locoregional disease within the abdomen and thorax. Nevertheless, Jennings et al. [13] reported that skeletal system is frequently the first site of identifiable distant metastatic spread, and bone scan is recommended to exclude metastatic diseases before radical treatment of esophageal cancer. Most importantly, the majority of esophageal cancers in previous studies evaluating the role of bone scan [4,12,13] are esophageal adenocarcinoma, not esophageal squamous cell carcinoma. Gockel et al. [14] reported that these two histologic tumor types in esophageal cancer exhibit different behavior, and squamous cell carcinoma shows an earlier lymphatic spread and a worse prognosis compared to adenocarcinoma. To the best of our knowledge, a large series study evaluating the role of bone scan in esophageal squamous cell carcinoma is lacking. Correct detection of bone metastases in patients with esophageal squamous cell carcinoma is crucial for prognosis and selection of an appropriate treatment regimen. The aim of the present study was to evaluate the role of whole-body bone scan in patients with esophageal squamous cell carcinoma.

## Methods

### Patient selection

Between December 1999 and January 2008, 360 consecutive patients with biopsy- proven esophageal squamous cell carcinoma at Kaohsiung Chang Gung Memorial Hospital were retrospectively reviewed after excluding patients with synchronous or antecedent cancers other than esophageal squamous cell carcinoma. This study was approved by the Institutional Review Board of Chang Gung Memorial Hospital. The median age of these patients was 55 years (range 29–81 years). There were 351 men and 9 women. The median durations of follow-up were 1886 days (range 841–3600 days) for the 61 survivors and 395 days (range 24–3600 days) for all 360 patients. Upper gastrointestinal endoscopy was performed to establish the diagnosis, appearance, extent and level of the tumor. After the diagnosis of esophageal squamous cell carcinoma, spiral computed tomography (CT) of chest (from neck to upper abdomen) and/or endoscopic ultrasonography (EUS) were performed for clinical staging. After intravenous administration of contrast, CT of the chest was performed in 5-mm sections. EUS was carried out with a radial scanning videoechoendoscope at 12 MHz (Olympus UM-2R). Some of the patients did not receive EUS because of the esophageal stenosis or distant metastases were already detected by CT of the chest. Bronchoscopy was used to verify tracheoesophageal (T-E) fistula in selected patients. After CT scan or/and EUS were available, pretreatment clinical staging was determined according to the 7^th^ American Joint Committee on Cancer (AJCC) staging system. Among these 360 patients, there were 33 patients with AJCC 7^th^ stage I disease, 86 patients with AJCC 7^th^ stage II disease, 201 patients with AJCC 7^th^ stage III disease, and 40 patients with AJCC 7^th^ stage IV disease.

**Figure 1:**
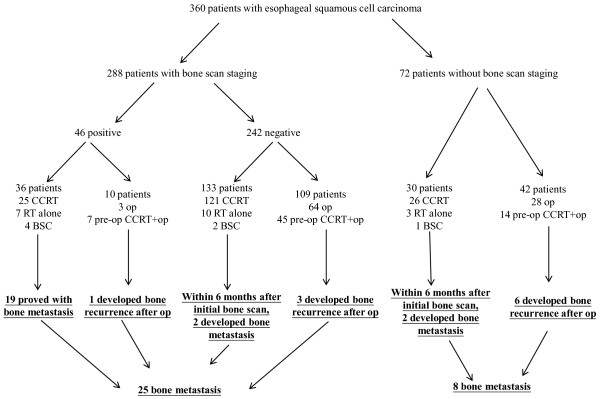
** Patient enrollment and study flow diaphragm.** CCRT, concurrent chemoradiotherapy; RT, radiotherapy; BSC, best supportive care; Op, operation; Pre-op CCRT + op, preoperative concurrent chemoradiotherapy followed by operation.

Of these 360 esophageal squamous cell carcinoma patients, 288 patients received bone scan during pretreatment staging, and 72 patients did not receive bone scan during pretreatment staging (Figure 1). Sensitivity, specificity, positive predictive value, and negative predictive value of bone scan in patients with esophageal squamous cell carcinoma were determined according to the results of these 288 patients.

Of these 360 patients, surgery was performed in 161 patients including 119 patients with preoperative bone scan and 42 patients without preoperative bone scan. Among these 161 patients receiving surgery, 133 patients had stages II + III disease, including 99 patients with preoperative bone scan and 34 patients without preoperative bone scan. All patients undergoing surgery had a radical esophagectomy with cervical esophagogastrostomy or Ivor Lewis esophagectomy with intrathoracic anastomosis, two-field lymphadenectomy, reconstruction of digestive tract with gastric tube and pylorus drainage procedures. Bone recurrence-free survival and overall survival were compared in all 161 patients and 133 stages II + III patients, respectively.

### Bone scan

99mTc-labelled methylene diphosphonate was used for whole-body bone scan. Bone scans were interpreted by an experienced nuclear medicine physician according to standard clinical practice, using intensity, configuration, location, and number of foci of abnormal radiotracer activity. Bone scan was considered positive for bone metastasis if radiotracer activity in the lesion was greater than that in normal bone, and the abnormal findings were multiple and asymmetric. Bone scan was considered negative for bone metastasis if there was no scintigraphic abnormality, or if there was a reasonable explanation of those scintigraphic findings for benign causes (osteoarthritis, osteomalacia, traumatic insults), or equivocal findings. If positive bone scan was noted, further investigation was performed using X-rays, computed tomography (CT) scans, magnetic resonance imaging (MRI) scans, radiologically guided bone biopsy or a combination of techniques.

### Confirmation of bone metastases

Patients were confirmed to have bone metastases if any of the following criteria were present: positive bone biopsy or radiographic confirmation by other imaging modalities such as X-rays, CT scans, and MRI scans. For patients with equivocal findings during initial radiological examination, further radiological follow-up within 6 months is necessary to confirm bone metastasis. For patients receiving esophagectomy, the bone metastasis events were recorded until death or the last follow-up. Bone recurrence was defined as bone metastasis detected as the first site of recurrence after esophagectomy or detected within 1 month of first site of recurrence after esophagectomy [7,13]. For patients who did not receive esophagectomy, the bone metastasis events were recorded within 6 months of initial bone scan. Therefore, a negative bone scan was considered as false negative if confirmed bone metastasis occurred after esophagectomy in patients with surgery or within 6 months of initial bone scan in patients without surgery.

### Statistical analysis

Statistical analyses were performed using the SPSS 17 software package. Sensitivity, specificity, positive predictive value, and negative predictive value were calculated using the classical method. The chi-square test, Fisher’s exact test, and student *t* test were employed to compare data between the two groups. Overall survival (OS) was calculated from the date of diagnosis to death as a result of all causes. Bone recurrence-free survival is computed from the time of surgery to the bone recurrence. To calculate the bone recurrence-free survival, patients who died without evidence of recurrence or recurrence other than bone were censored. The Kaplan–Meier method was used for univariate survival analysis, and the difference between survival curves was tested by a log-rank test. In a stepwise forward fashion, parameters with P values < 0.05 at univariate level were entered into Cox regression model to analyze their relative prognostic importance. However, as component factors of 7^th^AJCC staging system, 7^th^ T stage and 7^th^ N stage were not introduced in multivariate analyses. For all analyses, two-sided tests of significance were used with P < 0.05 considered significant.

## Results

### Sensitivity, specificity, positive predictive value, and negative predictive value in patients with esophageal squamous cell carcinoma

Of the 360 esophageal squamous cell carcinoma patients, whole-body bone scan was performed in 288 patients, and 119 of them received esophagectomy. Of the 119 patients receiving esophagectomy, 4 patients developed bone metastasis at 69 days, 104 days, 263 days, and 638 days respectively after esophagectomy (Figure 1). Among the 288 patients with whole-body bone scan, there were 23 patients with AJCC 7^th^ stage I disease, 69 patients with AJCC 7^th^ stage II disease, 165 patients with AJCC 7^th^ stage III disease, and 31 patients with AJCC 7^th^ stage IV disease (Table 1). Of the 72 patients without whole-body bone scan staging, 42 patients received esophagectomy, and bone metastasis occurred in 6 patients at 70 days, 94 days, 226 days, 253 days, 363 days, and 913 days respectively after esophagectomy (Figure 1).

**Table 1:** Sensitivity, specificity, PPV, and NPV of bone scan in 288 patients with esophageal squamous cell carcinoma

**7**^**th**^**AJCC Stage**	**Bone metastasis**	**Abnormal bone scan finding**	**Sensitivity**	**Specificity**	**PPV**	**NPV**
All (n = 288)	25	46	80.0% (20/25)	90.1% (237/263)	43.5% (20/46)	97.9% (237/242)
Stage I (n = 23)	0	2	-(0/0)	91.3% (21/23)	0% (0/2)	100.0% (21/21)
Stage II (n = 69)	4	10	75.0% (3/4)	89.2% (58/65)	30.0% (3/10)	98.3% (58/59)
Stage III (n = 165)	13	24	76.9% (10/13)	90.8% (138/152)	41.7% (10/24)	97.9% (138/141)
Stage IV (n = 31)	8	10	87.5% (7/8)	87.0% (20/23)	70.0% (7/10)	95.2% (20/21)

PPV, positive predictive value; NPV, negative predictive value.

Among 288 patients receiving bone scan, bone scan was positive in 46 patients and negative in 242 patients. Positive bone scan was significantly associated with T3 + 4 disease (P = 0.023, data not shown) and stage IV disease (P = 0.017, data not shown). Patients with positive bone scan had significantly (P = 0.006, data not shown) worse overall survival than those with negative bone scan. The median overall survivals were 248 days and 434 days in patients with positive and negative bone scan, respectively. The 3-year overall survival rates were 13% and 29% in patients with positive and negative bone scan, respectively.

Of the 242 patients with negative bone scan, 109 patients received esophagectomy. Of the 109 patients who received esophagectomy, 3 patients developed bone recurrence at 69 days, 263 days, and 638 days after esophagectomy. Of the 133 patients who did not receive esophagectomy, 2 patients developed bone metastasis within six months of initial negative bone scan (Figure 1).

Of the 46 patients with positive bone scan, 19 patients had bone metastases confirmed by X-rays in 1 patient, CT scans in 6 patients (pelvic CT in 2 patients, brain CT in one patient, and chest CT in 3 patients), MRI scans in 6 patients, and positive bone biopsy in 6 patients, respectively. In the remaining 27 patients, 10 patients received esophagectomy, and 1 patient was confirmed to have bone metastases 104 days after esophagectomy (Figure 1).

Of the 360 esophageal squamous cell carcinoma patients, 33 patients were confirmed to have bone metastases eventually and 327 patients were not. (Figure 1) The median overall survivals of 33 patients with confirmed bone metastasis and 327 patients without confirmed bone metastasis were 219 days and 419 days, respectively (P < 0.001, data not shown). The 3-year overall survival rates of 33 patients with confirmed bone metastasis and 327 patients without confirmed bone metastasis were 3% and 27%, respectively.

The presence of positive bone scan as an indication of metastasis presented 80% sensitivity, 90.1% specificity, 43.5% positive predictive value (PPV), and 97.9% negative predictive value (NPV) (Table 1). Then we performed subgroup analysis according to the 7^th^ AJCC stage. In 23 patients with stage I esophageal squamous cell carcinoma of our series, there were no evidence of bone metastasis. The sensitivity, specificity, PPV, and NPV of bone scan in patients with stage II, III, and IV esophageal squamous cell carcinoma are shown in Table 1.

### Absence of preoperative bone scan is significantly associated with inferior bone recurrence-free survival

**Figure 2:**
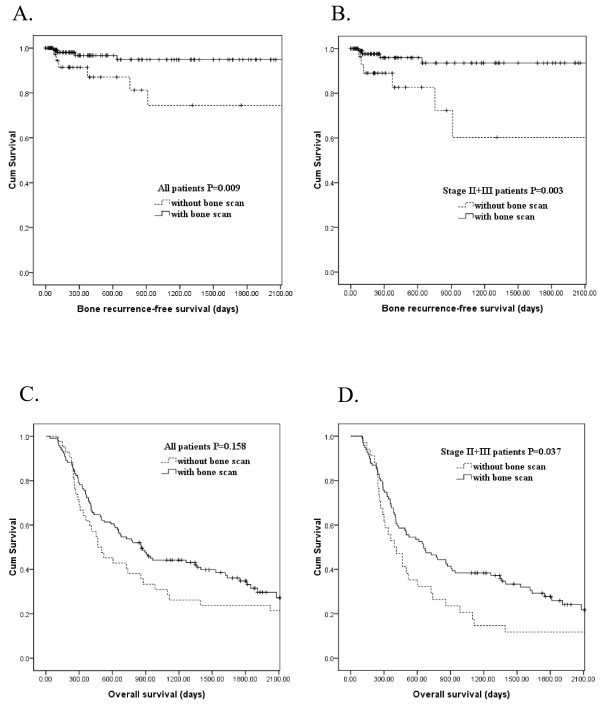
**Kaplan–Meier plots to predict bone recurrence-free survival according to the presence or absence of preoperative bone scan in all 161 patients (A) or 133 AJCC 7**^**th**^**stage II + III patients (B).** Kaplan–Meier plots to predict overall survival according to the presence or absence of preoperative bone scan in all 161 patients (**C**) or 133 AJCC 7^th^ stage II + III patients (**D**).

For patients with resectable esophageal squamous cell carcinoma, multiple treatment protocols including esophagectomy alone [7,15,16], preoperative concurrent chemoradiotherapy (CCRT) followed by esophagectomy [16,17], or definitive chemoradiotherapy [18,19] is preferred according to patient selection, physician preference, or comorbidity status. Evaluating the role of bone scan in different treatment protocols is difficult. However, if bone scan plays an important role in the staging of esophageal squamous cell carcinoma, it is reasonable to expect a significant reduction in the number of patients presenting with bone recurrence after esophagectomy. Therefore, we compared the bone recurrence-free survival between 119 patients with preoperative bone scan and 42 patients without preoperative bone scan. Four of the 119 patients with preoperative bone scan and 6 of 42 patients without preoperative bone scan developed bone recurrence. There was no significant difference between the two groups in baseline characteristics including age, sex, treatment protocol (esophagectomy alone or preoperative CCRT followed by esophagectomy), T stage, N stage, 7^th^ AJCC stage, and surgical margin. (Table 2) Univariate analysis (Table 3) showed that lymph node involvement (P = 0.014), 7^th^ AJCC stages III (P = 0.004), and absence of preoperative bone scan (P = 0.009, Figure 2A) were significantly associated with inferior bone recurrence-free survival after esophagectomy. In multivariate comparison, absence of preoperative bone scan (P = 0.012, odds ratio: 5.053, 95% confidence interval: 1.419-17.986) and 7^th^ AJCC stage III (P = 0.010, odds ratio: 7.891, 95% confidence interval: 1.630-38.190) represented the independent adverse prognosticator. The 3-year bone recurrence-free survival rates were 95% and 75% in patients with and without preoperative bone scan, respectively.

**Table 2:** Clinicopathologic features of 119 esophageal squamous cell carcinoma patients with preoperative bone scan and 42 esophageal squamous cell carcinoma patients without preoperative bone scan

**Parameters**		**No. of patients**		**P value**
		With bone scan before op (n = 119)	Without bone scan before op (n = 42)	
Mean age (range):54.34(29–77)		54.62 (37–77)	53.55 (29–66)	0.498
Sex	Male	115	41	1.000
	Female	4	1	
Protocol	Op	67	28	0.240
	Pre-op CCRT + op	52	14	
Primary tumor	T1 + 2	41	12	0.486
	T3 + 4	78	30	
Lymph node	Negative	55	20	0.876
	Positive	64	22	
7^th^ AJCC stage	I + II	60	20	0.755
	III	59	22	
Surgical margin	Positive	10	2	0.733
	Negative	109	40	

Op, operation; CCRT, concurrent chemoradiotherapy; х^2^ test, Fisher’s exact test, or *t* test was used for statistically analyzed.

**Table 3:** Results of univariate log-rank analysis of prognostic factors for bone recurrence-free survival in all patients (n = 161) receiving esophagectomy or stage II + III patients (n = 133) receiving esophagectomy

**Factors**	**No. of patients (All, n= 161)**	**Bone recurrence-free survival (All, n = 161)**		**No. of patients (Stage II + III, n = 133)**	**Bone recurrence-free survival (Stage II + III, n = 133)**	
		**No. of events**	**P value**		**No. of events**	**P value**
Bone scan before op						
Absent	42	6	0.009^*^	34	6	0.003^*^
Present	119	4		99	4	
Sex						
Male	156	10	0.480	129	10	0.475
Female	5	0		4	0	
Age						
<55y/o	88	4	0.417	71	4	0.584
>55y/o	73	6		62	6	
Tumor location						
Upper/middle	82	4	0.514	66	4	0.555
Lower	79	6		67	6	
Protocol						
Op	95	5	0.336	69	5	0.715
Pre-op CCRT + op	66	5		64	5	
T stage						
T1 + 2	53	2	0.160	25	2	0.757
T3 + 4	108	8		108	8	
N stage						
Negative	75	2	0.014^*^	47	2	0.100
Positive	86	8		86	8	
7^th^AJCC stage						
I + II	80	2	0.004^*^	52	2	0.037*
III	81	8		81	8	
Surgical margin						
Positive	12	0	0.505	10	0	0.604
Negative	149	10		123	10	

Op, operation; CCRT, concurrent chemoradiotherapy *Statistically significant.

In our series, we did not find bone metastasis in 23 patients with stage I esophageal squamous cell carcinoma, indicating that bone scan could be overlooked in patients with stage I esophageal squamous cell carcinoma in the future. Hence, subgroup analysis was performed to investigate the role of bone scan in patients with 7^th^ AJCC stages II + III esophageal squamous cell carcinoma. The bone recurrence-free survival between 99 stages II + III patients with preoperative bone scan and 34 stages II + III patients without preoperative bone scan were compared. There was no significant difference between the two groups in baseline characteristics including age, sex, treatment protocol (esophagectomy alone or preoperative CCRT followed by esophagectomy), T stage, N stage, 7^th^ AJCC stage, and surgical margin. (data not shown) By log-rank tests (Table 3), 7^th^ AJCC stages III (P =0.037) and absence of preoperative bone scan (P =0.003, Figure 2B) were significantly associated with inferior bone recurrence-free survival. Multivariate analysis showed that absence of preoperative bone scan (P = 0.009, odds ratio: 5.832, 95% confidence interval: 1.509-19.195) was the independent negative factor of bone recurrence-free survival. The 3-year bone recurrence-free survival rates were 93% and 60% in stages II + III patients with and without preoperative bone scan, respectively.

Later, we evaluate whether superior bone recurrence-free survival may convert into the overall survival advantage. We compared the overall survival between 119 patients with preoperative bone scan and 42 patients without preoperative bone scan. There was no significant difference in overall survival between 119 patients with preoperative bone scan and 42 patients without preoperative bone scan (P = 0.158, Table 4 and Figure 2C). Univariate analysis (Table 4) showed that T3 + 4 (P < 0.001), lymph node involvement (P < 0.001), 7^th^ AJCC stages III (P < 0.001), and positive surgical margin (P = 0.006) were significantly associated inferior overall survival. The median overall survivals were 860 days and 467 days in patients with and without preoperative bone scan, respectively. The 3-year overall survival rates were 44% and 31% in patients with and without preoperative bone scan, respectively. Then, we compared the overall survival between 99 stages II + III patients with preoperative bone scan and 34 stages II + III patients without preoperative bone scan. Univariate analysis (Table 4) showed that absence of preoperative bone scan (P = 0.037, Figure 2D), lymph node involvement (P < 0.001), 7^th^ AJCC stages III (P < 0.001), and positive surgical margin (P < 0.001) were significantly associated with inferior overall survival. In multivariate comparison, absence of preoperative bone scan (P = 0.029, odds ratio: 1.603, 95% confidence interval: 1.048-2.450), 7^th^ AJCC stage III (P = 0.002, odds ratio: 1.926, 95% confidence interval: 1.262-2.937), and positive surgical margin (P < 0.001, odds ratio: 3.937, 95% confidence interval: 1.953-7.937) represented the independent adverse prognosticators for overall survival in 133 stages II + III patients. The median overall survivals were 658 days and 395 days in stages II + III patients with and without preoperative bone scan, respectively. The 3-year overall survival rates were 38% and 21% in stages II + III patients with and without preoperative bone scan, respectively.

**Table 4:** Results of univariate log-rank analysis of prognostic factors for overall survival in all patients (n = 161) receiving esophagectomy or stage II + III patients (n = 133) receiving esophagectomy

**Factors**	**No. of patients (All, n = 161)**	**Overall survival (All, n = 161)**		**No. of patients (Stage II + III, n = 133)**	**Overall survival (Stage II + III, n = 133)**	
			**No. of events**		**P value**	**No. of events**	**P value**
Bone scan before op						
Absent	42	35	0.158	34	32	0.037*
Present	119	79		99	73	
Sex						
Male	156	113	0.064	129	104	0.075
Female	5	1		4	1	
Age						
<55y/o	88	63	0.820	71	59	0.161
>55y/o	73	51		62	46	
Tumor location						
Upper/middle	82	54	0.439	66	48	0.472
Lower	79	60		67	57	
Protocol						
Op	95	61	0.049*	69	53	0.347
Pre-op CCRT + op	66	53		64	52	
T stage						
T1 + 2	53	26	<0.001*	25	17	0.113
T3 + 4	108	88		108	88	
N stage						
Negative	75	39	<0.001*	47	30	<0.001*
Positive	86	75		86	75	
7^th^AJCC stage						
I + II	80	43	<0.001*	52	34	<0.001*
III	81	71		81	71	
Surgical margin						
Positive	12	10	0.006*	10	10	<0.001*
Negative	149	104		123	95	

Op, operation; CCRT, concurrent chemoradiotherapy *Statistically significant.

## Discussion

Among 288 patients with bone scan during pretreatment staging in the present study, bone metastasis was confirmed in 19 (6.6%) patients. Previous studies [13,20,21] showed that the incidence of bone metastasis in esophageal cancer during pretreatment staging was 4-9%. In the present study, 10 (6.2%) of 161 patients receiving esophagectomy developed bone recurrence. In the previous studies, [6,13,22,23] 5-9% patients developed bone recurrence after esophagectomy. Our results are similar to previous studies.

The results of bone scan were negative in 242 patients (84.0%), and only 5 patients of them developed subsequent bone metastases. The negative predictive value was high, and was not influenced by advanced stage (stages III and IV). However, the positive predictive value was low, especially in early-stage patients. Of the 46 patients who showed positive bone scan, 26 (56.5%) patients did not have bone metastasis, and in stage II patients, the positive predictive value was only 30.0%. It is important, therefore, the interpretation of bone scan need to take into consideration the context of clinical symptoms as well as any possible reasonable explanation for benign causes and the positive findings should be confirmed by further examinations such as X-rays, CT scans or MRI scans.

In our study, we did not find bone metastasis in 23 patients with stage I esophageal squamous cell carcinoma with a median follow-up of 1442 days, indicating that bone scan could be omitted in patients with stage I esophageal squamous cell carcinoma. Bhansali et al. [7] also described that no recurrence occurred from 15 esophageal squamous cell carcinoma patients with pT1 tumor. However, Mariette et al. [24] reported that for pT1 esophageal cancer, no recurrence occurred in 12 patients with tumor restricted to mucosa whereas recurrence was observed in 25 (31.8%) patients with tumor restricted to submucosa. But, the study from Mariette, et al. [24] included both esophageal squamous cell carcinoma and adenocarcinoma, and they described that distant recurrences occurred more frequently with adenocarcinoma. Therefore, we suggest that bone scan may be unnecessary for esophageal squamous cell carcinoma restricted to mucosa. For esophageal squamous cell carcinoma restricted to submucosa, further study is needed.

As previously described, there have been doubts about the routine use of bone scan in esophageal cancer. Furthermore, the majority of histology type in previous reports on the role of bone scan in esophageal cancer is adenocarcinoma, not esophageal squamous cell carcinoma. Large series data regarding the value of routine bone scan in esophageal squamous cell carcinoma are scant. Multiple treatment choices including surgery alone, preoperative CCRT followed by surgery, definite chemoradiotherapy, radiotherapy alone can be applied to patients with resectable esophageal squamous cell carcinoma according to patient selection, physician preference, or comorbidity status. Such hampered our evaluation on the role of bone scan in esophageal sqaumous cell carcinoma. However, if bone scan has an appreciable role in the initial staging of esophageal squamous cell carcinoma, it is logical to expect significantly superior bone recurrence-free survival in patients receiving esophagectomy. Indeed, in our univariate analysis, absence of preoperative bone scan correlated with inferior bone recurrence-free survival, and it remained prognostically independent in multivariate comparison, suggesting that bone scan should be routinely performed before esophagectomy in patients with esophageal squamous cell carcinoma. Additionally, we found that lymph node involvement was associated with inferior bone recurrence-free survival. Previous studies [7,24] also showed the correlation between lymph node involvement and distant organ recurrence. Our results further support previous findings.

In our series, bone metastasis was not detected in 23 stage I patients, indicating that bone scan could be omitted in stage I patients in the future. Hence, we perform subgroup analysis to evaluate the role of bone scan in stages II + III patients. In our univariate and multivariate analysis, absence of preoperative bone scan was significantly associated with inferior bone recurrence-free survival in stages II + III patients. We also found that absence of preoperative bone scan significantly correlated with inferior overall survival in stages II + III patients. It suggests that preoperative bone scan is a valuable tool in patients with esophageal squamous cell carcinoma, especially in patients with advanced stages.

Our study has important limitations. First, our results are based on the retrospective analysis. The retrospective design of this analysis further justifies the conclusion that a prospective study in the future is needed to define our findings. Second, most of the patients in this study did not have fluorodeoxyglucose positron emission tomography (FDG-PET) as comparison because it is not routinely supported by Taiwan’s health-insurance system. Third, the patients in the present study were staged based on the CT of the chest or/and EUS. The bone scan findings were not integrated into staging. Therefore, the disease staging in the preset study may be understaged.

## Conclusions

In conclusion, our study revealed that absence of preoperative bone scan is significantly associated with inferior bone recurrence-free survival. Therefore, we suggest that whole-body bone scan should be performed before esophagectomy in patients with esophageal squamous cell carcinoma, especially in patients with advanced stages.

## Competing interests

The authors declare that they have no competing interests.

## Authors' contributions

Study concepts: SHL. Study design: SHL and HIL. Data acquisition: SHL, HIL, WTH and WYT. Data analysis and interpretation: YCH and WCL. Statistical analysis: CTL. Manuscript preparation: SHL. Manuscript editing: HIL. Manuscript review: YCH and WCL. All authors read and approved the final manuscript.

## Pre-publication history

The pre-publication history for this paper can be accessed here:

http://www.biomedcentral.com/1471-2407/12/328/prepub
